# Correlation Between Heart Rate Variability and Agility Scores of Elite Badminton Players: A Pilot Study

**DOI:** 10.7759/cureus.58267

**Published:** 2024-04-14

**Authors:** Dobson Dominic, Sneha Thirugnana Sambandam, Harshavardhini Anburaj, Narayanaswamy Gopalakrishnan

**Affiliations:** 1 Sports Medicine, Saveetha Medical College and Hospitals, Saveetha Institute of Medical and Technical Sciences, Chennai, IND; 2 Cardiology, Saveetha Medical College and Hospitals, Saveetha Institute of Medical and Technical Sciences, Chennai, IND

**Keywords:** performance enhancement, autonomic nervous system, hrv, athletes, badminton

## Abstract

Introduction

Heart rate variability (HRV) indexes the autonomic nervous system, and HRV values are found to be higher in elite badminton players. Since an athlete’s agility has a direct influence on badminton sporting performance, this study will analyze the correlation between HRV and agility.

Aim

The study’s primary aim is to analyze the correlation between HRV and agility scores of elite badminton players.

Methodology

Ten elite badminton players who are currently participating at the state and national levels were recruited for the study. The study’s participants were aged between 18 and 21 years, had a body mass index (BMI) of less than 22.9 kg/m^2^, were currently training 10-12 sessions of badminton per week (120-180 minutes per session), and had no comorbidities, injuries, or illnesses.

For a duration of 14 days, a cross-sectional study design was utilized to evaluate the badminton players. Participants were tested in two blocks; each block consisted of five days of HRV and agility testing (Southeast Missouri [SEMO] agility test) followed by a break for two days. Higher agility performance was reflected by a lower SEMO agility test score.

Results

Data were analyzed using IBM SPSS Statistics for Windows, Version 27.0 (IBM Corp., Armonk, NY). HRV and agility scores had a negative correlation, as indicated by the two-tailed Spearman correlation analysis (*r*_s_(8) = -0.82, *P* < 0.01).

Conclusions

The results showed that HRV and agility scores are highly correlated in elite badminton players. The results indicate that higher HRV values lead to better agility performance. Future studies need to be conducted on a large scale to evaluate the correlation in a diverse population.

## Introduction

Badminton is a racquet sport that comprises complex upper-body and lower-body movements [1]. It was first introduced in Olympics in the year 1992 and has since gained much popularity. As an opponent-based sport, it requires swift multidirectional movements in reaction to the shuttlecock [1,2]. Excelling in the sport requires training in the strength, power, agility, speed, and stability components of the athlete [3-10]. This paper focuses on the agility component of professional badminton players.

Heart rate variability (HRV) is a measure of time variations in the beat-to-beat interval of the heart [11]. As the autonomic system regulates HRV, analysis of HRV indicates the functioning of the sympathetic and parasympathetic systems [11,12]. HRV is influenced by variations of factors such as gender, age, sporting level of the athlete, psychological and physical state, and other environmental factors [12]. Furthermore, the autonomic nervous system also influences the agility of an individual. A six-week progressive plyometric training program improved the agility scores of badminton players aged 20 to 24 years according to a study by Irawan [13,14].

Enhancing performance through evidence-based training principles proves crucial in sports development [15-17]. A previous study conducted by Singh et al. on state-level badminton players showed that agility as a physical characteristic significantly correlated with badminton performance, reiterating that improving the agility component can lead to improvement in badminton sporting performance [18,19]. Since the components of agility are regulated by the autonomic nervous system, a hypothesis was formulated that HRV and agility could be correlated in badminton players [20,21].

HRV monitoring is becoming a key tool in monitoring and prescribing optimal training load for athletes [22,23]. This pilot study aims to investigate the relationship between elite badminton players' HRV and agility performance. To the best of the authors' knowledge, no prior research has examined the relationship between badminton players' agility and HRV. The results provide a clearer understanding to sports medicine physicians, coaches, and athletes about the role of HRV on agility in badminton players. Furthermore, the results provide a basis for future research on integrating HRV-based badminton training to improve the agility performance of badminton players.

## Materials and methods

Participants

Ten elite badminton players defined as those who are currently participating at the state and national level were recruited for the study. The study comprised individuals who were between the ages of 18 and 21, male and female, had a body mass index (BMI) of <22.9 kg/m^2^, were engaged in 10-12 badminton training sessions per week (lasting 120-180 minutes each), and had no illnesses, injuries, or comorbidities. Participants were recruited from badminton clubs. Ethical clearance was obtained from the Saveetha Medical College and Hospital Institutional Ethics Committee (ref no. 014/05/23/IEC/SMCH) before the commencement of the research. Every participant was given an information sheet about the study post that they signed an informed consent form, stating their willingness to take part in the pilot study. The participants were not involved in the research's design, conduct, reporting, or dissemination plans.

The study was conducted at the Department of Sports Medicine and Sports Sciences at Saveetha Medical College and Hospitals, SIMATS, Chennai, India. Participants were recruited for the study from May 2022 to July 2023, and the study was conducted during August 2023.

Instruments

Biosignal Plux Explorer was used to measure heart rate variability in all the participants. It is a wireless data acquisition system that uses electrocardiogram sensors to record HRV. HRV was recorded while maintaining homogeneity in the testing environment for both the groups at both the pre- and post-intervention testing to minimize the influence of confounding factors [24]. Pereira et al. established the validity of Biosignal Plux in a study conducted in the year 2023 [25]. The root mean square of successive differences between normal heartbeats (RMSSD) value of the time domain index obtained from Biosignal Plux software was used for HRV analysis. BMI was calculated using a weighing scale and measurement tape, while agility scores were recorded using a stopwatch. 

The Southeast Missouri (SEMO) agility test, which is a validated agility test widely used in research studies, was used to measure agility [26]. A study conducted by Kirby in the year 1971 established the validity and reliability of this test [26]. The test involved setting up four cones in a rectangular manner measuring 3.7 and 5.7 m in dimension [26]. The athlete had to perform sidestepping, diagonal backward running, and forward sprinting to complete the test. The movements performed during this test were very similar to the badminton sports movements. The stopwatch was started during the first sidestep, and the test was deemed complete when the athlete returned to the starting cone.

Design

All the participants underwent a pre-participation evaluation before the start of the study. They were screened for any medical or surgical injury or illness that prevented them from taking part in the study. HRV analysis included time-domain analysis of HRV (RMSSD value), while body composition analysis included measurement of BMI.

To evaluate the badminton players over 14 days, a cross-sectional study design was implemented. Participants were tested in two blocks. Each block consisted of five days of HRV and agility testing followed by a break of two days. The participant’s daily HRV score was recorded in a supine position before the warm-up session. Post recording their RMSSD values for one minute, they were asked to warm up for 10 minutes. Following this, they were asked to perform two trials of the SEMO agility test, and the better of the two timings was recorded as the final score. A lower SEMO score showed that the athlete was able to complete the test faster, which indicated a higher level of agility performance. The RMSSD values obtained on the agility testing days (10 days) were used for analysis.

Statistical analysis

Statistical analysis was conducted using IBM SPSS Statistics for Windows, Version 27.0 (IBM Corp., Armonk, NY). Normality analysis was conducted using the Shapiro-Wilk test and histograms to compare the age and BMI values of the 10 badminton players. To determine the relationship between the agility and HRV scores, Spearman correlation analysis was utilized. The *P*-value was considered significant at 0.05.

## Results

**Figure 1:**
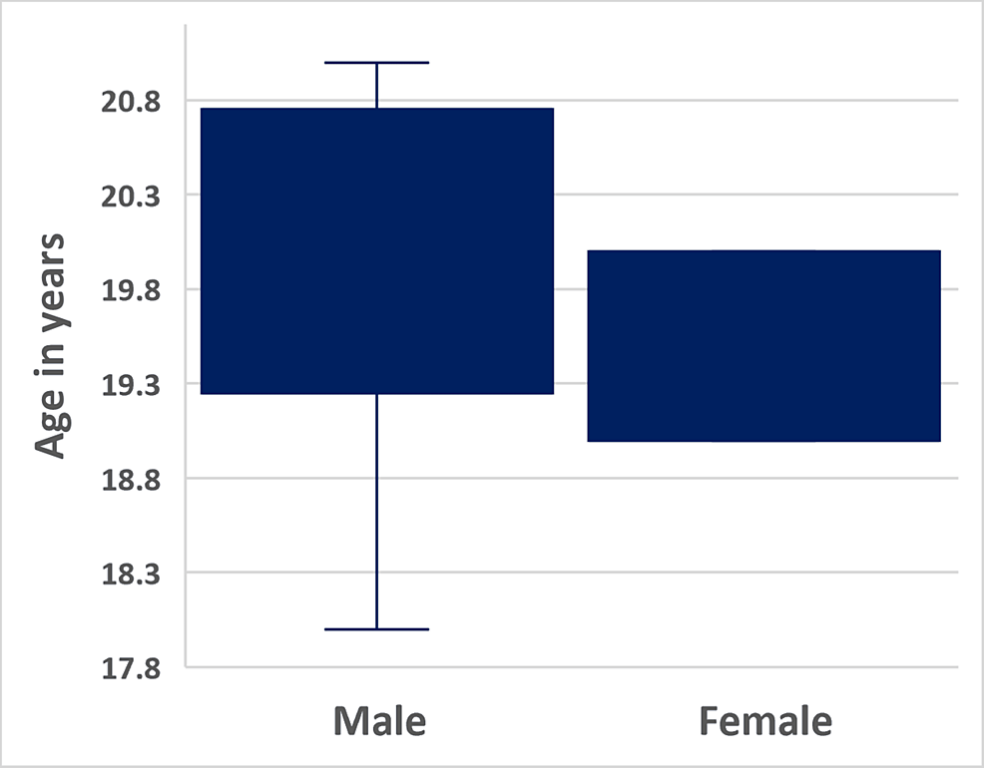
The mean age of the badminton players categorized according to gender. The *X*-axis shows the grouping as male and female, and the *Y*-axis shows the mean age of the badminton players represented in years.

All 10 badminton players completed the study, and there was no loss of follow-up during the testing period. Of the 10 badminton players, six players were male while four players were female (Figure 1). Shapiro-Wilk analysis showed that the badminton player’s age was not normally distributed, while the BMI values were parametric (Table 1). Six of the badminton players were playing the sport at the national level, while four were playing at the state level.

**Table 1:** Demographics of the population. SD, standard deviation; CI, confidence interval; BMI, body mass index

Variable	Mean (SD)	Lower bound (95% CI)	Upper bound (95% CI)
Age	19.70 (+/-0.95)	19.02	20.37
BMI	18.26 (+/-1.68)	17.06	19.46

**Figure 2:**
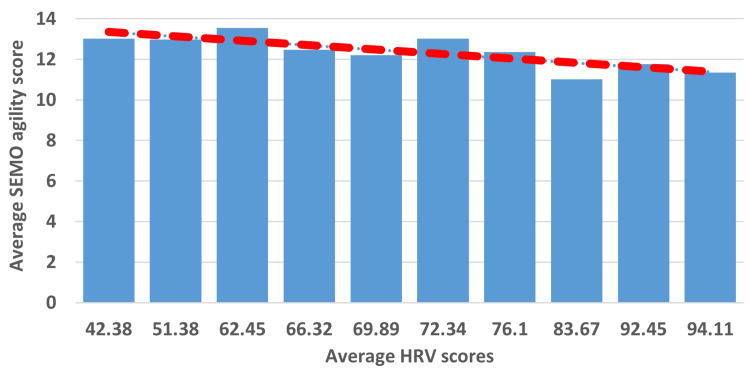
Average values of each participant’s HRV score plotted against their average SEMO agility scores over the testing period. The *X*-axis indicates the average RMSSD scores recorded for each badminton player during the testing period. The *Y*-axis represents the average SEMO agility score recorded for each player. The red line indicates the linear trend in the values. The results of the Spearman correlation analysis demonstrated a negative correlation (*r*_s_(8) = -0.82, *P* < 0.01) between the two variables. RMSSD, root mean square of successive differences between normal heartbeats; HRV, heart rate variability; SEMO, Southeast Missouri

The average score of the daily HRV recorded during the testing period and the average score of the SEMO agility score obtained during the same period were used for analysis. The results of the two-tailed Spearman correlation analysis showed a negative correlation between the two variables (HRV and agility scores), *r*_s_(8) = -0.82, *P* < 0.01 (Figure 2). Further analysis using the *t*-test showed that the SEMO agility scores significantly increased over the 14-day testing period in five of the 10 badminton players (13% improvement; *P* < 0.05). On observation of the values obtained from the other players, the authors found a mixed pattern of change in SEMO agility scores over the testing period.

## Discussion

The study aimed to explore the correlation between HRV and agility in elite badminton players. The findings of this study revealed that HRV is inversely proportional to agility scores, indicating that higher HRV values lead to greater agility performance. The findings have additional ecological validity because of the utilization of the SEMO agility test, which is intended to replicate movements unique to badminton [26]. The significant increase in agility scores highlights even more how important agility training is to improving badminton players' performance. According to correlation values categorized by Sugiyono, correlation values are defined as very weak if values are less than 0.199, weak if they fall between 0.20 and 0.399, medium from 0.40 to 0.599, strong from 0.60 to 0.799, and very strong from 0.80 to 1.00 [27]. The correlation value in this study was -0.82, indicating a significant and very strong negative correlation (*P *< 0.01) between HRV values and agility scores.

The elite badminton players who took part in this study were both male and female, showing a heterogeneous pattern of change in their SEMO agility scores throughout the 14-day testing session. Some athletes showed significant progress, whereas other players' performances were variable. The need for individualized training methods in badminton is highlighted by this diversity, which reveals the unique nature of athletes' reactions to training inputs [14,18]. A previous study conducted by Ravé et al., which explored the correlation between perceived physical fitness (PPF)and RMSSD scores in soccer players showed a moderated correlation between the two variables [28]. Although the study investigated the relationship between PPF and RMSSD, it did not investigate the correlation between HRV and agility [28].

In addition, the results also showed that the SEMO score improved in five players over the 12-day testing period highlighting the principle of specificity in sports training. The improvement in scores could be caused by the familiarization of the SEMO agility in the later sessions [14]. Research by Kuo et al., which demonstrated that a 12-week badminton footwork training program improved the ability and agility of badminton players, supports this theory [7]. Furthermore, an analysis of the results from the badminton players who did not display a significant increase showed that on the day they obtained lower scores during the terminal sessions, their HRV values were also low. This further highlights the primary hypothesis of this study that there is a correlation between HRV and agility.

This pilot study is notable since it's the first study to inquire into the correlation between badminton players' agility and HRV. The results indicate that HRV is an important determinant of agility performance in elite badminton players. According to a study done in the year 2016 by Kiss et al., athletes had substantially higher HRV values than healthy nonathletic subjects [29]. The results from the study indicate that sports training leads to short-term adaptations in the autonomic nervous system of an individual as a determinant of their sporting performance [29]. The results of this paper are also in accordance with the results of the study conducted by Kiss et al. showing that when the athlete’s HRV values were high, their agility scores were also elevated [29].

Limitations

This was a pilot study conducted to analyze the correlation between HRV and agility in elite badminton players, but due to the narrow age group of the participants, the results may not be representative of the entire population. The participants were selected from a particular region, which is another limitation of the study. Furthermore, the sample size is small; hence, future studies can be conducted to find the correlation on a large scale.

Recommendation

The generalizability and validity of the findings of this study would be strengthened by larger and more varied cohort studies in the future, along with the inclusion of control groups and longitudinal designs. Future studies can also focus on using HRV-guided badminton training methods to improve agility performance in badminton players.

## Conclusions

This study presents evidence that agility scores are better in badminton players with higher HRV values. The results showed that HRV and agility scores are highly correlated in elite badminton players. Future studies need to be conducted on a large scale to evaluate the correlation in a diverse population. Nevertheless, this study could serve as a basis for sports medicine physicians, athletes, and coaches in factoring HRV as an indicator of the agility performance of badminton players.
